# Strategies for tonal and atonal musical interpretation in blind and normally sighted children: an fMRI study

**DOI:** 10.1002/brb3.450

**Published:** 2016-03-22

**Authors:** Coral Guerrero Arenas, Silvia S. Hidalgo Tobón, Pilar Dies Suarez, Eduardo Barragán Pérez, Eduardo Castro Sierra, Julio García, Benito de Celis Alonso

**Affiliations:** ^1^ Escuela Nacional de MúsicaUniversidad Nacional Autónoma de MéxicoMéxicoDFMexico; ^2^ Departamento de ImagenologíaHospital Infantil de México Federico GómezMéxicoDFMexico; ^3^ Departamento de FísicaUniversidad Autónoma Metropolitana, Campus IztapalapaMéxicoDFMexico; ^4^ Department of RadiologyFeinberg School of Medicine ‐ Northwestern UniversityChicagoIllinois; ^5^ Facultad de Ciencias Físico ‐ MatemáticasBenemérita Universidad Autónoma de PueblaPueblaMexico

**Keywords:** Anterior prefrontal cortex, atonal, blind, BOLD, connectivity, correlation, dorsal posterior cingulate cortex, fMRI, functional, music, tonal, Wernicke's

## Abstract

**Introduction:**

Early childhood is known to be a period when cortical plasticity phenomena are at a maximum. Music is a stimulus known to modulate these mechanisms. On the other hand, neurological impairments like blindness are also known to affect cortical plasticity. Here, we address how tonal and atonal musical stimuli are processed in control and blind young children. We aimed to understand the differences between the two groups when processing this physiological information.

**Results:**

Atonal stimuli produced larger activations in cerebellum, fusiform, and temporal lobe structures than tonal. In contrast, tonal stimuli induced larger frontal lobe representations than atonal. Control participants presented large activations in cerebellum, fusiform, and temporal lobe. A correlation/connectivity study showed that the blind group incorporated larger amounts of perceptual information (somatosensory and motor) into tonal processing through the function of the anterior prefrontal cortex (APC). They also used the visual cortex in conjunction with the Wernicke's area to process this information. In contrast, controls processed sound with perceptual stimuli from auditory cortex structures (including Wernicke's area). In this case, information was processed through the dorsal posterior cingulate cortex and not the APC. The orbitofrontal cortex also played a key role for atonal interpretation in this group.

**Discussion:**

Wernicke′s area, known to be involved in speech, was heavily involved for both groups and all stimuli. The two groups presented clear differences in strategies for music processing, with very different recruitment of brain regions.

## Introduction

Understanding the plasticity effects that underpin brain physiological processes is of widespread interest. This knowledge can be used to help improve recovery drugs or rehabilitation procedures in people who are born with a disability such as blindness, or who have acquired it during their lifetime. Music is known to modulate cortical plasticity and improve not just musical abilities, but also others not related to music (Bailey and Penhune 2012), (Watanabe et al. 2008). Musical training is easy and inexpensive to implement, and is therefore a very interesting plasticity model to use and study. Furthermore, musical stimulation in children is much more interesting than in adults, as neural plasticity is greatest in the early years (Habib and Besson 2009; Penhune 2011).

Several neuroimaging modalities have been applied to study musical integration in the brain. The most common have been functional magnetic resonance studies using its blood oxygenation level‐dependent (BOLD) contrast (Ducreux et al. 2003), positron emission tomography (PET) (Zatorre et al. 1994), magnetoencephalography (Tervaniemi et al. 1999), and electrophysiology (Itoh et al. 2005). These studies have focused on describing the areas which were activated by a given stimulus at a given time point. The explosion of results with resting‐state techniques has opened the door of connectivity studies of BOLD low‐frequencies (i.e., (Luo et al. 2012) (Kay et al. 2012) (Fauvel et al. 2014). These works have shown that, in addition to the areas known to be activated during hearing (thalamus, primary auditory cortex, and Wernicke's area in the temporal lobe), music may activate other regions such as the putamen, premotor dorsal cortex, primary motor cortex, and supplementary motor cortex (Grahn and Brett 2007) (Bengtsson et al. 2009; Grahn 2009). Task‐related BOLD studies showed how prefrontal, frontal, and parietal cortex were also activated during rhythm perception. Other authors (Peretz and Zatorre 2007) have observed activations in these same regions, by musicians when performing discrimination tasks. These same authors described a response in the right anterolateral part of Heschl's gyrus, which is a cortical region dedicated to auditory processing during tone analysis (Peretz and Zatorre 2007). Foster et al. (Foster and Zatorre 2010) showed that transposing musical pieces of different pitches to the original one was associated with intraparietal sulcus activity.

Physiological differences between blind individuals and normally sighted controls have been found in the past. First, several works using fMRI on rats have shown that the unaffected sensory systems occupy the areas designated to an “affected” sense in the cortex (Albieri et al. 2014), (Bengoetxea et al. 2012). Bengoetxea et al. showed activations in the occipital cortex in response to sensory and auditory stimuli. This was supported by (Klinge et al. 2010), who showed that visual regions were used by blind human volunteers to process an auditory cue. Shu et al. (Shu et al. 2009) showed decreased efficiency in connectivity for blind versus control groups in a diffusion tensor imaging study. The most affected areas were the occipital medial and superior gyri, as well as the cuneus. There was also a correlation between the inferior frontal lobe and the occipital lobe that was missing or ineffective for the blind group. In a voxel‐based morphometry study, (Noppeney et al. 2005) demonstrated recruitment of occipital areas when processing tactile, auditory, and complex cognitive tasks. (Bedny et al. 2010) did not show activation in visual MT/MST regions for motion, but did for pitch changes. The authors argued for the existence of a multimodal function in these regions, which only appeared when they had never been used to process images. Finally, (Jiang et al. 2009) observed, using MRI, that visual cortex in the blind was thicker than in controls. They argued that this was due to the lack of pruning of nonuseful neurons during the developmental periods of life.

Differences in musical interpretation between blind and control individuals have also been found. Many of these differences are due to plasticity. Yabe et al. (Yabe and Kaga 2005) showed that blind volunteers had a better auditory spatial ability than individuals with partial blindness. Audio‐motor feedback was found to replace vision, to calibrate auditory space in blind individuals. Thus, compensatory plasticity mechanisms were enhanced by increased processing of proprioceptive and vestibular information with the auditory spatial input (Lewald 2002). It is also known that blind individuals perform much better in auditory tasks than normally sighted controls (Hugdahl et al. 2004). A study by Roder et al. (2000) showed that, during auditory processing of language, congenitally blind volunteers performed better than sighted people. This indicated that blind participants spoke faster than controls, and also suggested the possibility of cortical reorganization.

Even though musical interpretation in the blind has been studied in the past, to our knowledge a larger and more detailed study assessing functional connectivity and its changes (due to cortical plasticity) using fMRI is lacking. The objective of this study was to assess the differences in brain recruitment and connectivity when interpreting tonal and atonal music in normally sighted and blind pediatric populations. To this end, a BOLD‐fMRI experiment was performed to quantify BOLD areas activated, and the ROI‐to‐ROI correlations between them.

## Materials and Methods

### Participants

Bioethical guidelines from the Helsinki protocol were followed in this study of human subjects. Permission from the local ethics committee (Ethics committee from the Hospital Infantil del DF, Federico Gómez, HIFG from now on) was obtained to perform the study. Before enrolling in the study, all participants were informed of what to expect during the experiments, as well as the possible dangers of participating in it. After agreement, their legal guardians were asked to sign the corresponding consent forms. Twenty‐five pediatric participants were recruited for this study. Blind and control patients were randomly recruited from the imaging and pediatric departments of the HIFG. As this is the national reference hospital for pediatric care in the country, it reaches people of all socioeconomic backgrounds. This selection process gave no reason to believe that the study group was not representative of Mexican population, even though no thorough socioeconomic background or IQ matching was performed on volunteers. Ten volunteers were blind children and 15 were normally sighted controls. Ages varied between 5 and 6 years old (both sexes with prevalence of boys 60% over girls 40% in both groups). All participants were right‐handed, belonged to a functional family without a record of internal violence, did not know braille, and had no indicators of any neurological disorder. No volunteers had ever been trained in any kind of artistic discipline. The nature of the blindness in the blind group was congenital glaucoma (blind since birth). Volunteers from both groups were healthy during the study protocol. Trained medical doctors in the hospital, to ensure that volunteers fulfilled the inclusion criteria for this study, performed background health checks.

### Protocol

After standard preparation for an MR scanning session, volunteers were introduced into the scanner in the supine position, with headphones. Cushions between the head and the coil were added to avoid head motion. Headphones were used to receive instructions from scientists as well as the musical stimuli. After standard MR preparation, an anatomical scan followed by a BOLD‐fMRI sequence. Time duration of the first scan sequence was 3 min. The second scan sequence took 12 min 54 sec. The whole scanning session (which lasted for 15 min 54 sec) was then considered finished and patients were extracted from the scanner and their participation ended.

### Stimulation paradigm

The fMRI study was a task‐related paradigm. In it, two musical stimuli were randomly delivered to participants with silence periods in‐between. Both musical stimuli lasted for 12 sec, while silence periods lasted for 16 sec, allowing BOLD signal to reach baseline values. Both stimuli were repeated 10 times in a random fashion in each fMRI study.

The musical stimuli were both designed specifically for this study with *Sibelius* software (Avid Technology, Inc., Burlington, MA). These sounds did not belong to any sound database. Both stimuli were intended to be nonemotional and to this end, it was decided to use tonal (T from now on) and atonal stimuli (A from now on). Piano melodies in Do Major with a 4/4 compass, so timing would fit that of stimuli periods (12 sec), were used. The T stimuli used regular rhythms in a high pitch. For the A stimuli dissonant notes or errors (six dissonant notes per stimulus were used, making this stimuli atonal), no white noise was used in these silence periods between musical stimuli as it has been found to produce characteristic BOLD activations for some auditory stimuli (Lindemberg and Scheef 2007).

### Hardware

Scanning was performed in a 1.5 T Philips‐Intera Achieva scanner (Philips, Inc., Amsterdam, Netherlands). This system had a NOVA gradient system set (Copley 271 Dual, slew rate of 80 mT/m/ms and peak amplitude of 120 mT/m). A Philips SENSE head coil with multichannel technology was used (eight channels).

### MR sequences

As presented in the protocol section, two sequences were run. Anatomical information was obtained with a T_1_‐weighted gradient echo sequence, fast field echo (FFE), with repetition time (TR) = 307.81 ms, and echo time (TE) = 2.48 ms, flip angle = 80°, matrix = 640 × 640, in‐plane resolution = 0.36 × 0.36 mm^2^, thickness = 4 mm, and number of excitations (NE) = 4. This sequence used 35 axial slices (without gaps), which covered the whole brain of pediatric volunteers, including their cerebellum. It provided a field of view (FOV) of 23 × 23 × 14 cm^3^. After anatomical imaging, an fMRI‐BOLD sequence followed. Data was acquired with a T_2_*‐weighted gradient echo sequence (FFE). MR parameters in this case were TR = 3000 ms, TE = 35 ms, flip angle = 80°, matrix = 64 × 64, in‐plane resolution = 3.6 × 3.6 mm^2^, thickness = 4 mm, and NE = 1. The same FOV as in the anatomical case was covered, with 35 axial slices per volume. A total of 255 volumes per fMRI‐BOLD experiment were acquired. In order to allow for BOLD signal saturation, 9 sec of dummy scan acquisition were added at the start of the fMRI sequence. Also, every stimulation protocol started with a 16‐sec silent period which was not considered for image analysis later on. These parameters are the same ones used by this research group in previous studies (Alonso et al. 2014),(Platas‐Neri et al. 2015).

### Image analysis

#### Software

Image analysis was performed in Matlab (The Mathworks Inc.) using SPM (SPM8; http://www.fil.ion.ucl.ac.uk/spm, Natick, MA) software for the construction and analysis of the general linear model (GLM) in fMRI analysis (first‐ and second‐level analysis). Before that, the DPARSFA toolbox was used for batch preprocessing of images. The REST toolbox was used for graphical presentation (http://www.restfmri.net). Finally, the CONN‐fMRI connectivity toolbox v.12.k was used for the connectivity study (http://www.nitrc.org/projects/conn).

#### Preprocessing

Batch preprocessing was performed with the DPARSFA toolbox. First, DICOM data was transformed into the Analyze format used by SPM8. A slice time correction was then performed (ascending and centered on the 18th slice). Realignment followed using the standard SPM routines, which use a least‐squares approach with six parameters (rigid body). Authors required motion to be <1.5 mm (half a voxel) and 1.5° of rotation during the whole fMRI acquisition. Framewise displacement power (FD) (Power et al. 2013) and FD Van Dijk analyses (Van Dijk et al. 2012) were also performed to assess that no motion effect was confounding results (FD was always kept under 0.5). All volunteers satisfied these four requirements and no subjects were rejected due to motion in this study. Standard segmentation using DARTEL sequences followed, whereby CSF, white, and gray matter masks were built for each individual. Information obtained from the first two masks was eliminated, as they have no physiological relevance to this study. Only information obtained from applying masks to gray matter on individual participants was used down the analysis pipeline in the BOLD‐fMRI and in the correlation study. Data were then normalized to an EPI template in MNI coordinates. Gray matter was then further segmented into the regions of the Anatomical Automatic Labeling (AAL) atlas (Tzourio‐Mazoyer et al. 2002). All voxels in a given region were then used to calculate the average time course for the area. Finally, the normalized data was smoothed to eliminate noise due to scanning and preprocessing artifacts, and to fulfill the statistical requirements of the GLM. This was done with a 10.8 × 10.8 × 12.3 mm^3^ kernel (three times the size of a voxel).

#### BOLD‐FMRI analysis

With the data preprocessed, SPM software was used to perform first‐ and second‐level analysis. To this end a GLM was built for the two musical stimuli and the two groups. At the first level, a contrast was built for each possible combination (tonal (T) for blind (B), atonal (A) for B, T for controls (C), and A for C). Second‐level analysis created the following four contrasts: blind T versus A, control T versus S, tonal B versus C, and atonal B versus C. With all of these contrasts, a significance threshold of *P* < 0.05 with false discovery rate (FDR) correction was applied.

Results were then overlaid on the MNI template using REST software. With this software, BOLD activations could be associated with their corresponding Brodmann and AAL regions (including brain hemisphere information), and the size of activated clusters calculated. Quantification of the BOLD results was performed using the total number of significant voxels obtained from the images of the group study.

#### Connectivity analysis

Finally, connectivity calculations for the four second‐level contrasts were performed using the CONN‐fMRI connectivity toolbox. Here, ROI‐to‐ROI correlations were calculated with the same significance threshold as before (*P* < 0.05, FDR corrected). This software allows comparisons between ROI′s from the AAL atlas (same as in the BOLD‐fMRI study). For this analysis, the average BOLD time series for a given region was used. This information was automatically obtained with this software from the preprocessed data of the previous section (information just from the masked gray matter). For presentation purposes, images were overlaid on coronal slices of an EPI template with the seed ROI represented in black and the connected regions in red. When a single region was correlated with more than one other region, the term network was used.

## Results

This study was divided into two parts. In the first section, we obtained BOLD‐fMRI volumes for the different contrasts. The second section concerned the results of a connectivity (correlation) study on the same contrasts.

**Figure 1:**
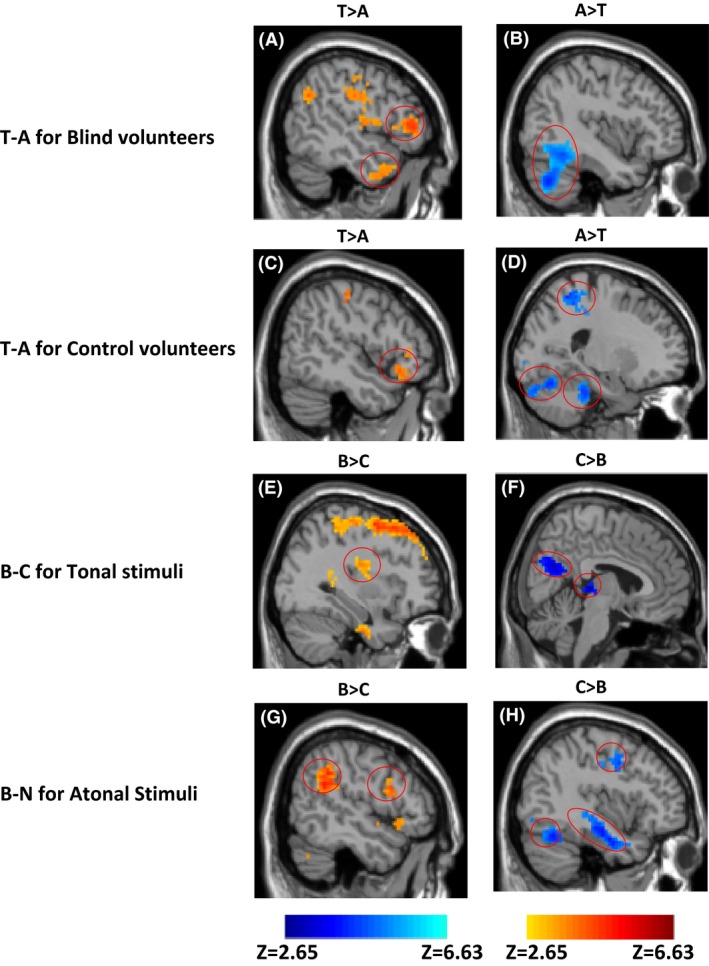
Examples of blood oxygenation level‐dependent (BOLD)‐fMRI results. BOLD activations were overlaid on sagittal anatomical images. The description of the contrast used appears in the left column, while the direction of contrast appears indicated by labels on top of figures (i.e., the second row is the tonal vs. atonal contrast for control volunteers exclusively). Threshold values of 2.65 < Z < 6.63 were used to build these images. A pseudocolored bar showing the significance of results can be found under the panels. (A) Shows activation in the inferior temporal and frontal lobes. Trends of activation also appear in the rolandic operculum and the supramarginal gyrus. (B) Shows activation in the left fusiform and also in cerebellar structures. (C) Shows activation in the right inferior frontal cortex. (D) Shows activation encircled in red figures in the cerebellum (region 4 and 6), as well as in the precentral region of cortex, all in the left hemisphere. (E) Shows significant results in the insula (inside red circle), and also in the frontal cortex and the fusiform cortex. (F) Shows results in the cuneus and the right thalamus. (G) Shows representations in the superior temporal lobe, as well as the frontal inferior operculum. (H) Shows characteristic left cerebellum, fusiform, and precentral activations.

Results for the first part of the study can be found in Table 1 and in Figure 1. Figure 1 shows some examples of BOLD activations in relevant regions, that is, the first row presents the contrast T versus A for B participants with activations in the inferior temporal lobe as well as the frontal lobe (Fig. 1A), when T was “stronger” than A. In contrast, Figure 1B shows activations in the cerebellum which were stronger for A when compared to T in the B group. Table 1 shows the volumes of BOLD activations for each contrast, in the same manner as Figure 1. Data is presented showing the Broadmann and AAL areas in which activations were found, together with their coordinates in MNI space (point of maximum BOLD signal), laterality, and the number of voxels active in each region (volume).

**Table 1:** Results from the BOLD study

BA	AAL	Volume	X	Y	Z	BA	AAL	Volume	X	Y	Z
Tonal‐atonal for blind subjects (T > A)						Tonal‐atonal for blind subjects (T < A)					
48	Rolandic operculum L	30	−58	1	8	42	Temporal superior R	38	55	−42	22
48	Supramarginal L	38	−63	−26	22	39	Temporal medial R	44	35	−60	22
45	Fronto inferior L	24	−47	36	1	37	Fusiform L	18	−41	−57	−13
32	Anterior cingulate L	44	−10	42	8	23	Precuneus R	38	4	−56	22
20	Temporal inferior L	20	−48	8	−34	19	Occipital superior R	14	22	−76	22
11	Fronto medial orbital L	38	−10	53	−13	X	Cerebellum Crus 2 L	60	−28	−75	−41
11	Fronto superior R	6	24	57	1						
6	Superior motor L	44	−3	6	57						
6	Precentral L	39	−30	−25	57						
3	Postcentral R	51	46	−24	57						
X	Medium cingulate R	21	3	−21	50						
Tonal‐atonal for control subjects (T > A)						Tonal‐atonal for control subjects (T < A)					
48	Insula R	7	41	−2	11	37	Temporal inferior R	28	39	−63	−6
47	Frontal medial orbital R	5	38	40	−13	37	Temporal medial L	19	−50	−71	8
40	Parareal inferior L	30	−47	−49	57	37	Cerebellum Crus 1 L	14	−43	−65	−27
38	Frontal inferior orbital R	5	48	24	−13	37	Fusiform R	18	36	−51	−20
32	Fronatal superior R	35	13	28	50	30	Cerebellum 4 L	8	−20	−31	−27
21	Temporal medial L	7	−50	−52	−6	18	Cerebellum 6	45	−7	−67	−20
20	Temporal inferior R	5	56	−45	−13	18	Lingual R	14	21	−89	−13
11	Anterior cingulate R	6	5	36	−6	10	Frontal superior med L	20	−5	63	15
8	Superior motor area L	45	−8	24	50	7	Postcentral L	8	−23	−49	57
6	Fronatal superior R	16	24	−3	50	5	Precuneus L	11	−5	−46	57
Blind‐controls for tonal stimuli (B > C)						Blind‐controls for tonal stimuli (B < C)					
48	Temporal Superior R	6	47	0	−7	37	Fusiform L	15	−32	−51	−13
48	Temporal Superior L	6	−51	−2	0	37	Fusiform R	9	32	−62	−6
48	Insula R	4	33	−13	21	27	Thalamus R	13	10	−27	1
20	Fusiform R	6	−7	−16	−21	20	Temporal merdial L	15	−56	−33	−13
						19	Fusiform L	13	−36	−74	−13
						18	Cuneus R	21	11	−70	22
						17	Calcarine R	20	3	−63	15
6	Precentral R	4	58	6	21	6	Occipital medial L	17	−27	5	43
6	Frontal superior R	12	15	−8	63	6	Frontal Superior L	6	−17	18	43
3	Postcentral R	6	40	−32	49	X	Cerebellum 10 L	20	−20	−31	−41
X	Cerebellum 8 R	4	7	−47	−56	X	Cerebellum Crus 2 L	25	−22	−78	−41
						X	Cerebellum Crus 1 L	23	−38	−60	−27
Blind‐controls for atonal stimuli (B > C)						Blind‐controls for atonal stimuli (B < C)					
48	Temporal superior R	4	46	−2	−6	37	Fusiform L	25	−41	−59	−20
48	Insula R	10	35	−10	22	37	Temporal medial R	23	41	−63	−3
44	Frontal Inferior operculum R	6	51	13	22	32	Frontal superior L	10	−20	17	44
38	Temporal superior pole R	4	49	18	−13	21	Temporal medial L	18	−56	10	−20
22	Temporal superior R	9	51	−48	22	20	Fusiform L	18	−39	−13	−20
20	Temporal inferior R	4	58	−19	−20	19	Occipital medial R	15	37	−79	1
20	Hippocampus R	4	30	−12	−15	18	Cuneus L	13	3	−80	29
9	Frontal medial R	4	35	13	50	10	Frontal superior L	19	−21	52	8
8	Frontal superior R	4	16	20	57						
6	Frontal superior R	7	16	−10	64	6	Precentral L	17	−28	−1	43
3	Parietal inferior R	9	43	−33	50	X	Cerebellum 4_5 L	30	−21	−30	−34
						X	Vermis 7	23	−4	−66	−27
						X	Thalamus R	15	10	−21	1

This table presents the blood oxygenation level‐dependent (BOLD) volumes which were statistically significant under stimulation of both kinds of sounds, for the four contrasts studied in this project. Data are presented under a contrast description in the following manner. First, Brodmann area (BA) activated; second, region activated according to Anatomical Automatic Labeling (AAL) with an indication of the hemisphere in which the structure was found, left (L) or right (R); third, the volume of the BOLD activation (number of activated voxels). Finally, coordinates in MNI space of the maximum voxel inside the significant BOLD activation are given.

For the two first contrasts, in which T and A were compared (independently of the volunteer being B or C), the major BOLD findings were as follows. First, larger activations for A in cerebellum and fusiform areas (127 voxels vs. 0 voxels for cerebellum and 36 voxels vs. 0 voxels in fusiform). Second, T clearly induced larger activations in the frontal lobe than A (123 vs. 20 voxels). Third, both stimuli activated the temporal lobe, but activations were larger for the A stimuli (129 vs. 33 voxels). Finally, T‐activated larger areas for B in the motor cortex, pre‐ and postcentral gyri, rolandic operculum, anterior and medial cingulate, and supramarginal cortex. In contrast, A activated more for B in the precuneus and occipital cortex. For C, tonal stimuli activated more in the insula, superior motor cortex and parietal lobe. A activated more for C in the precuneus, and postcentral gyrus.

For the third and fourth contrasts, in which B and C were compared (independently of the type of stimuli), the major findings were the following. First, larger activations for C in cerebellum (98 vs. 4 voxels) and fusiform (10 vs. 4 voxels) regions than B volunteers. Second, no major differences in frontal lobe activation were found between groups (35 voxels for C vs. 33 for B). Third, a tendency for temporal lobe to be more activated in C than in B (56 for C vs. 33 for B). Finally, the B group showed unique representations in the insula (both contrasts), and the pre‐ and postcentral regions. C volunteers had unique representations in the cuneus, thalamus (both contrasts), and precentral gyrus.

**Figure 2:**
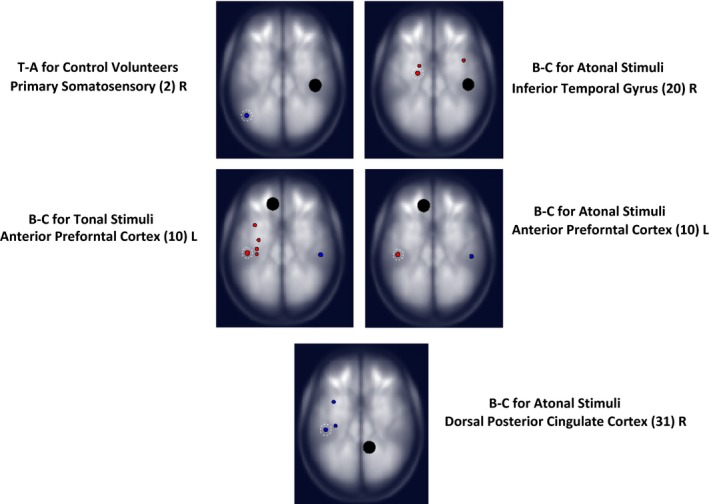
Examples of results from the connectivity study. This figure presents five examples of correlations which were significant (*P* < 0.05 false discovery rate corrected) in this study. The black points represent the reference Brodmann area region correlated with all the other regions. Its name and hemisphere appears in a label under the contrast description by each image. A red color indicates correlations which were stronger for the first group of the contrast. Blue color show the opposite effect.

Results for the connectivity study can be found in Figure 2 and Table 2. Figure 2 shows some significant correlations between ROIs for five different cases. The black dot in the images represents the seed region, and the red and blue dots represent correlated areas. The size of the dot represents the statistical strength of the correlation. Red indicated that the first group of the contrast dominates the second and blue the opposite. The top left panel shows a blue correlation, indicating stronger correlation for A than T between these regions for C volunteers. Table 2 shows the correlations for each contrast, with the added information of which hemisphere was connected (L and R for left and right), which Brodmann area (BA) they belong to (number in parentheses), and the statistical significance of the correlation (using Z values).

**Table 2:**
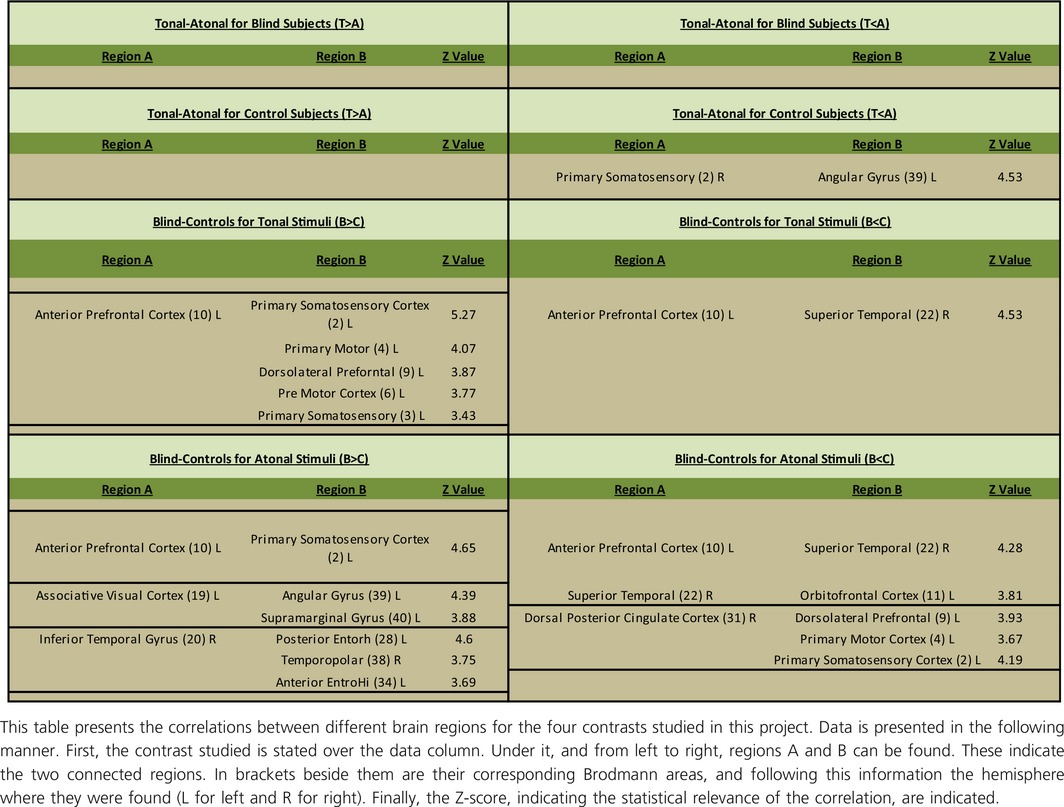
Results from the connectivity study

In general, similar connectivity was found when comparing T versus A for both groups (first two contrasts, independent of B or C group), pointing toward similar interpretation processes for these stimuli. The only exception was a correlation between the primary somatosensory area and the angular gyrus, found for A in the C group.

This trend was not followed in the B versus C comparisons in which large differences appeared. A strong correlation between anterior prefrontal cortex (APC) and superior temporal gyrus (in C volunteers) appeared for T and A musical interpretation. This network contrasted with the correlations found for the B individuals, in which the APC was linked to primary somatosensory and motor cortices, as well as the premotor cortex and the dorsolateral prefrontal cortex (DLPCC, Network 1a). This same network was limited to the primary somatosensory cortex for the A stimuli in B (Network 1b). The B group showed a network correlating associative cortex to the angular gyrus and the supramarginal gyrus (Network 2). A correlation of the inferior temporal gyrus to the temporopolar area and the anterior and posterior entorhinal cortex (Network 3) was also found for B volunteers for N. For the C group, a network correlating the dorsal posterior cingulate cortex (DPCC) was found, connecting to the DLPCC and the primary somatosensory and motor cortex (Network 4). In addition to these five networks, a strong correlation between the superior right temporal lobe and the orbitofrontal cortex (OFC) was found for C participants.

## Discussion & Conclusions

The results of this study may be summarized as follows. In the BOLD study, the cerebellum and fusiform regions played a big role for both groups, presenting large activations for A and the control group. Frontal lobe activations were strongly associated with both musical stimuli in both groups. Temporal activations were found for all the stimuli, but were larger for A and the control group. In the correlation study, differences appeared between the B and C groups. First, perception regions were heavily used by B volunteers (Networks 1a and 1b) to interpret the T stimuli. A similar network to 1a and 1b was found for the C group when interpreting A (Network 4), which excluded the frontal lobe. Finally, the B group had two independent networks (2 and 3) for the A stimuli, which were not present in C volunteers.

### Cerebellum and fusiform cortex activations

In a review by Petacchi and colleagues (Petacchi et al. 2005), it was shown that the cerebellum was activated in 73.3% of papers in which auditory stimulations were performed (fMRI or PET). The results of the meta‐analysis also pointed towards increased cerebral activation during increased sensory demand. Nevertheless, this still remains to be proven. Furthermore, the main activation in cerebellum found in this meta‐analysis was in the crus 1 region. This representation was independent of the rhythm or tone of the auditory stimulus. This led the authors to hypothesize that it was a region more involved with processing of sound cues than with perception of them (Bower 2002). The involvement of the cerebellum, not just in motion but in cognitive tasks, is nowadays very well‐supported (Stoodley et al. 2012).

Results from our BOLD study showed activations in crus 1 together with several other cerebellar activations like crus 2 (adjacent to crus 1) and cerebellum 4, 6. This applied for the A stimulus, but not for the T one. These results support the findings of Petacchi's meta‐analysis with respect to activation in the cerebellum, and specifically to crus 1. Furthermore, they suggest that larger activations would be present for sound cues that are perceptually more complicated. We could argue that the A stimulus was harder to interpret by both groups (B and C), and therefore larger cerebellar activations were regularly found for the A stimuli but not the T ones. Finally, the lack of activation for the T stimuli, in which rhythm and tones are present, supports the role of the cerebellum as a processing center for auditory stimuli, independently of the characteristics of the sound.

The fusiform cortex has conventionally been associated with the interpretation of faces and colors. Nevertheless, it has recently been linked with auditory stimuli and multisensory integration (Kawase et al. 2005). It is now known to be involved in attention changes, from visual to auditory stimuli (Shomstein and Yantis 2004). Lesions to this area allow for perception of sound, but not for its spatial localization (Clarke et al. 2002). The fusiform region was found to be related to background segregation in auditory processing when detecting the “richer” tones that accompanied a melody (Schmithorst 2005; Masayuki et al. 2011). Finally, B volunteers have presented activations due to speech in the fusiform gyrus (Hertrich et al. 2009).

Results from our BOLD study showed activations in the fusiform cortex, supporting the findings of other studies which used auditory cues. Activations appeared consistently for the A stimuli, but not the T ones. This might support both the processing role (segregation as stated by Masayuki et al.) and the integration of different stimuli role (Kawase et al.^2005^) of the region, when presented with extracomplicated sounds, that is, A rather than M. For the young child volunteers of this study, activations in the fusiform region were larger in C than B volunteers, partially contradicting findings from the Hertrich study. First, the fact that fusiform activation appeared for the blind group in two of the contrasts studied in the BOLD study, should rule out the possibility of a lack of enrollment of this area, or malfunction for these volunteers. Second, a possible source of this discrepancy could be the different type of stimuli presented to volunteers (speech vs. music), since it is known that they involve different brain networks for information processing (Zatorre et al. 1994). Still, the role of this region in tonal and atonal interpretation should be further analyzed.

### BOLD activations

For years the temporal lobe has been known to be the center of audition processing in healthy humans. Larger activations for the C group and for A were in line with the argument that A was a more complicated stimulus to interpret than M, and therefore required longer detection and larger processing areas to assess it. An interesting finding of this study would be that the C group showed the “standard” ability to recruit the traditionally auditory processing areas, while B volunteers used other mechanisms and strategies to process auditory inputs.

In a recent publication by (Flores‐Gutiérrez et al. 2013), relaxing and chaotic music was played to depressed patients and controls. The control group showed large activations in the frontal and temporal lobes for the chaotic music, but no activation in the frontal or inferior temporal lobes was seen for the relaxing music. The authors argued that, for the chaotic music, there is a physiological necessity to incorporate extraneuronal function (extrafrontal activations) to give a meaning to the sound being perceived. We have argued that A in this case is the most complicated of the two stimuli. It is with T that larger BOLD activations were detected in the frontal lobe. It could be argued that the extra neuronal activity found for T in the frontal lobe could correspond to emotional associations with the stimulus. This is supported by the larger BOLD activations found in the limbic system for music (medial and anterior cingulate), when compared to A.

### Different connectivity networks

Network 1a showed strong correlations between the left APC and the left primary somatosensory cortex for both stimuli. This correlation was extended to the motor cortex (primary motor and premotor), as well as the DLPF cortex, for the tonal stimuli. The APC has traditionally been involved in executive function, memory recall, and strategic analyses. Recently, it was also associated with retrieval of sound sequences (Rauschecker 2005), experience of pleasantness (Thakral et al. 2012), and much more relevantly, the integration of inputs of several sensory systems to achieve a conceptual interpretation of the environment (Paraskevopoulos et al. 2014), (Ramnani and Owen 2004). The latter seems to be the mechanism by which B volunteers were processing the tonal stimuli here. They used APC to integrate inputs from somatosensory and motor cortex. They also used its memory and retrieval of sound sequences to process music. This function of the APC was completed by the DLPFC and its cognition and memory functions, but especially task switching (Monsell 2003). As stated by several authors, blind individuals tend to use touch (somatosensory and motor inputs) to process their environment in normal day‐to‐day life. Therefore, they specialize in using this strategy, and use it to interpret other stimuli. Furthermore, the regions used for these stimuli are recruited (via cortical plasticity) to perform this function.

One could then ask, why do blind volunteers just have a fraction of Network 1a for atonal interpretation? Networks 2 and 3 appear as a possible complement for perception. Network 2 presents two correlations between Wernicke's area and the visual association cortex (V3, V4, and V5). Wernicke's area is known to be mainly involved in speech and word processing. Could this be an indication of a new tonal or atonal function for this area? We hypothesized that the involvement of the visual cortex as an example of a cortical plasticity phenomenon, in which blind volunteers used this cortex for a different function. The same argument could be applied to Network 3. Here, the inferior temporal gyrus, which is associated with visual processing, was used to process information from the entorhinal cortex (direction and perception of the environment) and the temporopolar cortex with an unknown function.

The correlation found for the control group between APC and BA 22 (Network 1b) could be expected, as the former is a secondary association auditory cortex. Nevertheless, it corresponds to Wernicke's area (as for Network 2). This again supports the role of this area in tonal processing.

When comparing Network 1a, which B used to interpret the tonal stimuli, and Network 4, which was used by the C group to interpret atonal, it can be seen that the main difference was the absence of the APC and its substitution by the DPCC. The perception integration and memory retrieval function of APC was substituted for by the DPCC and its little known list of functions. The most relevant of them would be retrieval of autobiographical memories, planning of the future, supporting internally directed cognition, and regulating the focus of attention (Leech and Sharp 2014). Control participants seem to also use several perceptive inputs for N, but instead of integrating the information in the APC they detect them and change attention from one to other without processing it further. The processing was apparently performed by an OFC correlation to the superior temporal cortex. The OFC has been proven to have a role in intuition processes, in which the content of a stimulus (music) had to be detected only with partial information (missing or distorted parts of the music stimuli). This would fit perfectly well with the N stimulus we delivered here, and once again indicated a more “standard” processing mechanism in controls which was not seen in the blind group.

## Conflict of Interest

None declared.
